# Simvastatin Combined with CpG Enhances the Immunogenicity of the H9N2 Inactivated Vaccine

**DOI:** 10.3390/vetsci12090855

**Published:** 2025-09-04

**Authors:** Yan Ma, Jiaxi Zhu, Zuchen Song, Lina Jiao, Ruihong Yu, Zheng Wang, Zhimin Zhang, Jiaguo Liu, Zhenguang Liu

**Affiliations:** 1 Institute of Traditional Chinese Veterinary Medicine, College of Veterinary Medicine, Nanjing Agricultural University, Nanjing 210095, China; 2 College of Life Sciences, Longyan University, Longyan 364012, China; 3 Academy for Advanced Interdisciplinary Studies, Nanjing Agricultural University, Nanjing 210095, China

**Keywords:** Simvastatin, CpG, H9N2, vaccine, adjuvant

## Abstract

The H9N2 virus has severely harmed the livestock and bird farming industry. Currently, it is mainly prevented through vaccination immunization. However, conventional vaccines often fail to induce durable immune responses and long-lasting immunoprotection. In this research, we used Simvastatin (Sim) and CpG as adjuvants for the H9N2 inactivated vaccine to evaluate the vaccine’s immunogenicity in chickens. We evaluate vaccine immunogenicity through antibody testing, T lymphocyte phenotyping, and RNA-sequencing analysis. The results indicated that the Sim + CpG/H9N2 formulation significantly enhanced specific IgY and hemagglutination inhibition (HI) antibody titers. It also increased the proportions of CD4^+^ T cells and CD8^+^ T cells, promoted immune organ development, and stimulated the formation of germinal centers. RNA-sequencing analysis revealed that Sim + CpG/H9N2 vaccination significantly upregulated immune-related genes, which were enriched in pathways associated with stress response activation, immune cell recruitment, and inflammatory signaling. Overall, these findings demonstrate that Sim + CpG/H9N2 markedly enhances the immunogenicity of the inactivated H9N2 vaccine and provides new insights into the application of vaccine adjuvants for improved immune protection.

## Simple Summary

This study explored the effects of combining Simvastatin and CpG as adjuvants in an inactivated H9N2 vaccine. After two rounds of vaccination, the results showed that the combination significantly boosted both specific IgY antibody titers and Hemagglutination Inhibition (HI) antibody titers. Additionally, it enhanced the populations of CD4^+^ and CD8^+^ T cells in peripheral blood mononuclear cells (PBMCs), thereby strengthening both humoral and T cell-mediated immune responses. Importantly, this combination exhibited no signs of biotoxicity to major organs. RNA-sequencing analysis revealed that the combination of Simvastatin and CpG promotes immune activation and immune cell recruitment. These findings suggest that this adjuvant combination effectively overcomes the limitations of using a single adjuvant, eliciting a broader and more potent immune response. This approach provides valuable insights for the design and development of more effective influenza vaccines.

## 1. Introduction

The H9N2 avian influenza virus (AIV) genome consists of eight RNA segments [1]. Its surface glycoprotein hemagglutinin (HA) exhibits a binding preference for α-2,6-sialic acid receptors (human-type receptors) [2], while the neuraminidase (NA) is of the N2 subtype. First isolated in turkeys in the United States in 1966, the virus mainly includes low pathogenic avian influenza virus (LPAI) and highly pathogenic avian influenza virus (HPAI) [3]. LPAI is one of those, and it is known for spreading fast and having a low death rate. It primarily causes respiratory and gastrointestinal symptoms in poultry, leading to reduced body weight and decreased egg production, which causes huge economic losses for the poultry industry [4].

At present, vaccination still remains the number one most effective way to prevent the H9N2 influenza virus [5]. The major categories of vaccines available on the market are attenuated live vaccines and inactivated vaccines. The latter is widely used because of its high safety. However, inactivated vaccines alone have issues such as weak cellular immune activation and short-lived immunity, and cannot induce sufficient immune protection [6]. Therefore, the incorporation of appropriate adjuvants is necessary to enhance the immunogenicity and efficacy of these vaccines.

Adjuvant-mediated immunomodulation is an effective strategy to improve vaccine efficacy and prolong immune persistence [7]. Traditional adjuvants, such as oil emulsions and aluminum-based compounds, have been shown to enhance the humoral immune response but are generally ineffective at inducing robust cellular immunity [8,9]. Therefore, the development of novel adjuvants represent a promising approach to enhancing vaccine-induced immune responses [10]. The mevalonate pathway is crucial for lipid metabolism and immune function. Research indicates that blocking key enzymes in this pathway disrupts the production of downstream metabolites. This disruption, in turn, impairs the membrane localization of Rab5/7 proteins and hinders endosome maturation [11]. This influences antigen processing and presentation, thereby modulating immune responses. Simvastatin, a well-known inhibitor of the mevalonate pathway, is drawing growing interest for its potential as an immunomodulatory [12]. CpG oligodeoxynucleotides (CpG ODNs) activate both innate and adaptive immunity via avian Toll-like receptor 21 (TLR21), significantly enhancing antibody production and cellular immune responses [13]. Despite challenges related to sequence optimization and production costs, CpG ODNs possess a favorable safety profile and exhibit strong Th1-polarizing capacity, making them promising candidates for use in the prevention and control of major avian epidemics, such as avian influenza and Newcastle disease.

In this study, we selected simvastatin and CpG ODNsas combined adjuvants for an inactivated H9N2 vaccine to investigate their synergistic effects on vaccine efficacy. Our results demonstrated that the Sim + CpG/H9N2 formulation elicited robust humoral and cellular immune responses, along with an acceptable safety profile. Furthermore, potential signaling pathways and the expression of immune-related genes associated with Sim + CpG were analyzed through RNA-sequencing analysis of bursa of fabricius tissue. In conclusion, the combination of simvastatin and CpG ODNs exhibited a synergistic adjuvant effect greater than the sum of its parts (“1 + 1 > 2”), effectively overcoming the limitations of single-component adjuvants. This strategy induced a more comprehensive and potent immune response, offering new insights into the design and application of improved influenza vaccines.

## 2. Materials and Methods

### 2.1. Reagents and Materials

Reagents and materials included rabbit anti-chicken IgG(IgY)-HRP (Beijing Boaosen Biotechnology, Beijing, China), β-propiolactone (McLean Biochemical, Shanghai, China), TMB substrate solution and CCK8 detection kit (Yisen Biotechnology, shanghai, China), SPF chicken red blood cell suspension (Senbeijia Biotechnology, Nanjing, China), H9N2 avian influenza virus strain AIV1536 (China Center for Virus Culture Collection, Beijing, China), peripheral blood lymphocyte separation medium (Beijing Solabio Technology, Beijing, China), mouse anti-chicken monoclonal antibodies (CD3-FITC, CD4-PE, CD8-APC; Southern Biotech, Birmingham, AL, USA), simvastatin (Me Chem Express, Shanghai, China), and CpG ODN (synthesized by Sangon Biotech, Shanghai, China; and the CpG sequence is 5′-TCGTCGTTGTCGTTTTGTCGTT-3′).

### 2.2. Vaccine Preparation

The H9N2 virus (AV1563) was injected into 9 days old specific pathogen-free chicken embryos (Nanjing Special Giving Breeding Cooperative, Nanjing, China). The virus was harvested from the urine cavity, and its hemagglutination (HA) titer was determined. Viral samples with an HA titer of 2^8^ were inactivated using 0.05% β-propiolactone. The fully inactivated viral fluid was mixed with different adjuvants at a 1:1 ratio.

### 2.3. Animals and Immunization Programmes

The one-day-old Hyland Brown chickens were bought from the Nanjing Special Giving Breeding Cooperative. All animal husbandry practices were carried out in strict accordance with ethical guidelines for the welfare of laboratory animals.

One-day-old healthy chickens were reared under standard conditions for a period of time. Following exclusion of maternal antibody interference, chickens were randomly allocated to six experimental groups for immunization, including: Blank group, H9N2 group (250 µL H9N2), Alum/H9N2 group (5 mg aluminum hydroxide and 250 µL H9N2), Simvastatin/H9N2 group (1 mg simvastatin and 250 µL H9N2), CpG/H9N2 group (20 µg CpG and 250 µL H9N2), Simvastatin + CpG/H9N2 group (1 mg simvastatin, 20 µg CpG and 250 µL H9N2). Each chicken was intramuscularly injected with 500 µL of the respective vaccine formulation into the leg muscle, followed by a booster immunization on day 14.

### 2.4. Antibody Detection and Hemagglutination Inhibition Assay

At 21, 28, 35, and 42 days post-primary immunization, four chickens per group underwent random selection and wing vein blood collection for serum isolation.

To assess the H9N2-specific IgY antibody levels, the quantification of H9N2-specific IgY antibody levels in serum were performed by enzyme-linked immunosorbent assay (ELISA). The procedure is as follows: First, coat 4 units of inactivated H9N2 antigen onto a 96-well ELISA plate and incubate at 4 °C overnight for 20 h. Then, block with 5% skim milk at 37 °C for 2 h. Samples are incubated with diluted serum at 37 °C for 1 h, followed by anti-chicken IgY secondary antibody incubation at 37 °C for 1 h. Three PBST washes are conducted after each step. Lastly, add the substrate solution for color development, then add the stop solution, and read the absorbance at 450 nm using an enzyme-linked immunosorbent assay reader (OD450) [14,15].

For hemagglutination inhibition (HI) antibody titer determination, the collected serum was subjected to serial two-fold dilutions in 96-well microplates. An equal volume of H9N2 antigen (4 hemagglutination units) was added to each well, and the mixture was incubated at room temperature for 30 min. Then, 1% chicken erythrocyte suspension was added and incubated for another 30 min. Record the highest dilution of serum that completely prevents blood clotting as the HI titer.

### 2.5. Determination of the Proliferation Index of Peripheral Blood Lymphocytes

Twenty-one days after the first vaccination, collect blood samples from each group of chickens. Mix the collected anticoagulated blood with PBS in a 1:1 ratio and dilute. The diluted anticoagulated blood was slowly added to 15 mL centrifuge tubes containing lymphocyte separation solution. The upper layer in the centrifuge tube is the anticoagulated blood, and the lower layer is the lymphocyte separation solution. Centrifuge for 30 min at room temperature. Remove the intermediate cloudy white cell clumps, wash with PBS, and resuspend in RMPI 1640 medium containing 1% penicillin, 5% H9N2, and 10% fetal bovine serum (FBS). The resuspended lymphocytes were incubated at 37 °C for 24 and 48 h. The proliferation index of peripheral blood lymphocytes was determined using the CCK8 assay method.

### 2.6. Assessment of Peripheral T Lymphocyte Populations

Twenty-one days after the first vaccination, blood samples were taken from each group of chickens. Peripheral blood lymphocytes were isolated according to the method described in Section 2.5 above. The cells were then suspended in RPMI-1640 medium containing 1% penicillin, 5% H9N2 antigen, and 10% FBS. The cells were evenly spread across a 24-well plate and cultured at 37 °C for 48 h. After incubation, the cell suspension was centrifuged at 4000 rpm for 5 min, the supernatant was discarded, and the cell pellet was collected. The collection of cells were colored with Anti-CD3e-FITC, Anti-CD4-PE, and Anti-CD8-APC antibodies and analyzed by flow cytometry.

### 2.7. Quantitative Real-Time PCR (qPCR) Analysis

On day 7 after the initial immunization, the bursa of fabricius was collected from each group of chickens. On day 21, spleens and peripheral blood lymphocytes were collected. And they were harvested for RNA extraction. The extracted RNA was reverse-transcribed into cDNA and analyzed by real-time quantitative PCR (qPCR). The list of primers used is presented in Table 1.

### 2.8. RNA-Sequencing Analysis

Seven days after the primary vaccination, three chickens were randomly chosen, dissected and their bursa of Fabricius was obtained. Total RNA was withdrawn using the TRIzol conventional RNA extraction kit. RNA sequencing was conducted by Meiji Bio in Shanghai, China.

### 2.9. Determination of Immune Organ Index

At 21 days after the initial immunization, the body weight of each chicken was recorded. Then perform a necropsy on the chicken to obtain the bursa of Fabricius, thymus, and spleen. Then wash the tissue with PBS, weigh it, record it, and calculate the immune organ index. The formula for calculating the immune organ index is as follows:

Organ Index (g/kg) = Organ Fresh Weight (g)/Live Body Weight (kg)

### 2.10. Safety Assessment

On day 42 after the initial immunization, we collected lungs, livers, kidneys, spleens, and bursa of fabricius and fixed them in 4% paraformaldehyde. We then prepared H&E-stained sections and examined them under a microscope to evaluate vaccine safety by checking for signs of inflammatory infiltration or tissue damage.

### 2.11. Statistical Analysis

All data were analyzed using GraphPad Prism 9 software, and *p* < 0.05 was set as the significant difference and indicated as follows: * *p* < 0.05, ** *p* < 0.01, *** *p* < 0.001. The results were expressed as means ± *SEM*.

## 3. Results and Discussion

### 3.1. Sim + CpG/H9N2 Enhanced the Antibody Response of the H9N2 Inactivated Vaccine

**Figure 1:**
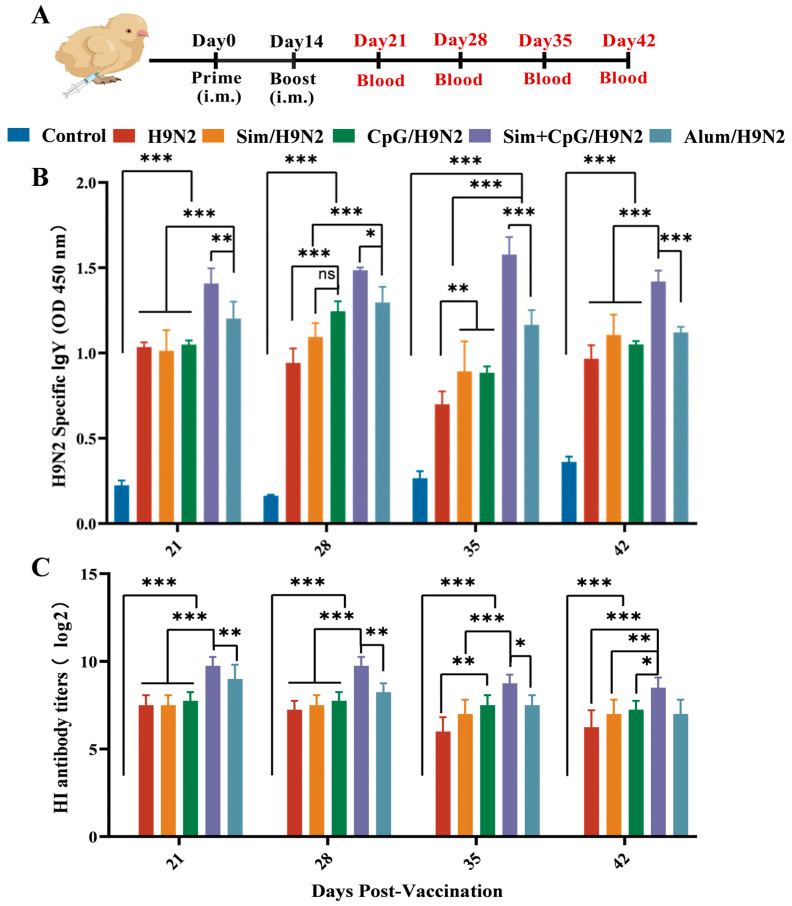
Antibody response induced by the H9N2 vaccine with adjuvants. (**A**) H9N2 vaccination schedule and sampling time points. (**B**) H9N2-specific IgY antibody titers. (**C**) H9N2-specific HI antibody titers. All data are presented as mean ± standard deviation. Statistical significance is defined as ns (no significance), * *p* < 0.05, ** *p* < 0.01, *** *p* < 0.001.

Antibodies are an important indicator for assessing vaccine immunity levels and are directly related to the long-term protectiveness of vaccines [16]. To fully assess the immunostimulatory potential of CpG and Simvastatin as adjuvants for the H9N2 inactivated vaccine, we collected serum after the initial immunization and tested them for specific IgY antibodies and HI titers (Figure 1A). As shown in the figure, when Simvastatin and CpG are used together as adjuvants for the H9N2 inactivated vaccine, they can significantly induce high levels of specific IgY antibodies, significantly higher than the H9N2 group and the Alum/H9N2 group (Figure 1B). This suggests that simvastatin combined with CpG can significantly enhance humoral immunity and boost the antibody response to the vaccine.

The production of hemagglutination-inhibiting (HI) antibodies is considered a marker of an effective influenza vaccine and an indicator of humoral immunity [17]. The protective HI threshold (≥4 log_2_) indicates antibody-mediated blockade of HA receptor binding, which is a critical mechanism for viral neutralization and transmission inhibition. In this research, the Sim + CpG/H9N2 group exhibited significant HI antibody titers over the 42-day period, confirming that simvastatin and CpG as adjuvants can effectively promote antibody production (Figure 1C).

These results suggest that using simvastatin and CpG as adjuvants significantly enhances the humoral immunity of the H9N2 vaccine. This effect is seen not only in IgY antibody titers but also in higher HI antibody levels, providing promising prospects for improving the efficacy of inactivated vaccines.

### 3.2. Sim + CpG/H9N2 Promotes the Cellular Immune Response of the H9N2 Inactivated Vaccine

Cell-mediated immunity is one of the core pillars of vaccine protection, especially for clearing intracellular infections, providing long-term memory, responding to pathogen mutations, and fighting cancer [18]. Among these, lymphocyte activation is central to initiating and regulating cell-mediated immune responses. It plays a crucial role throughout the immune defense process [19]. The proliferation and activation of lymphocytes form the foundation for vaccines to offer comprehensive, sustained, and mutation-resistant protection.

**Figure 2:**
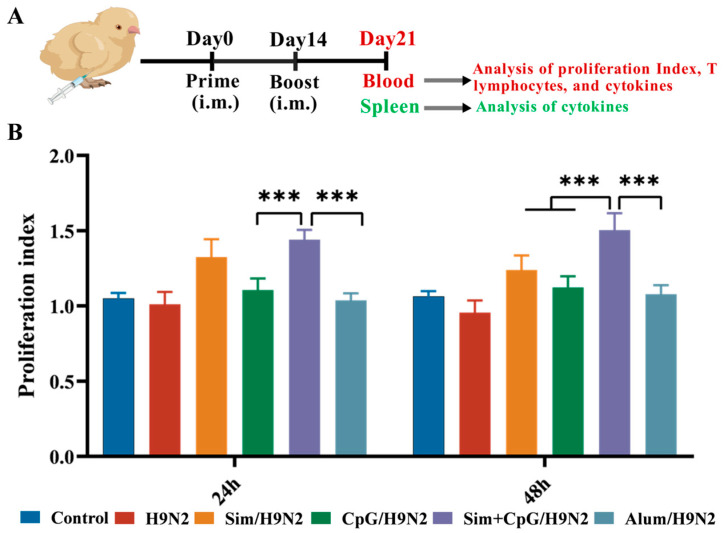
Cellular response induced by the H9N2 vaccine with adjuvants. (**A**) Immunization schedule and sampling timeline. (**B**) Peripheral blood lymphocyte proliferation index after antigen re-stimulation. All data are presented as mean ± standard deviation. Statistical significance is defined as *** *p* < 0.001.

To evaluate the effect of Sim + CpG on the cellular immune response to the H9N2 vaccine, peripheral blood lymphocytes (PBMCs) were isolated 21 days after primary immunization. The lymphocytes were then cultured in vitro with H9N2 antigen re-stimulation (Figure 2A). The results show that compared to other groups, Sim + CpG/H9N2 considerable promoted lymphocyte proliferation (Figure 2B).

**Figure 3:**
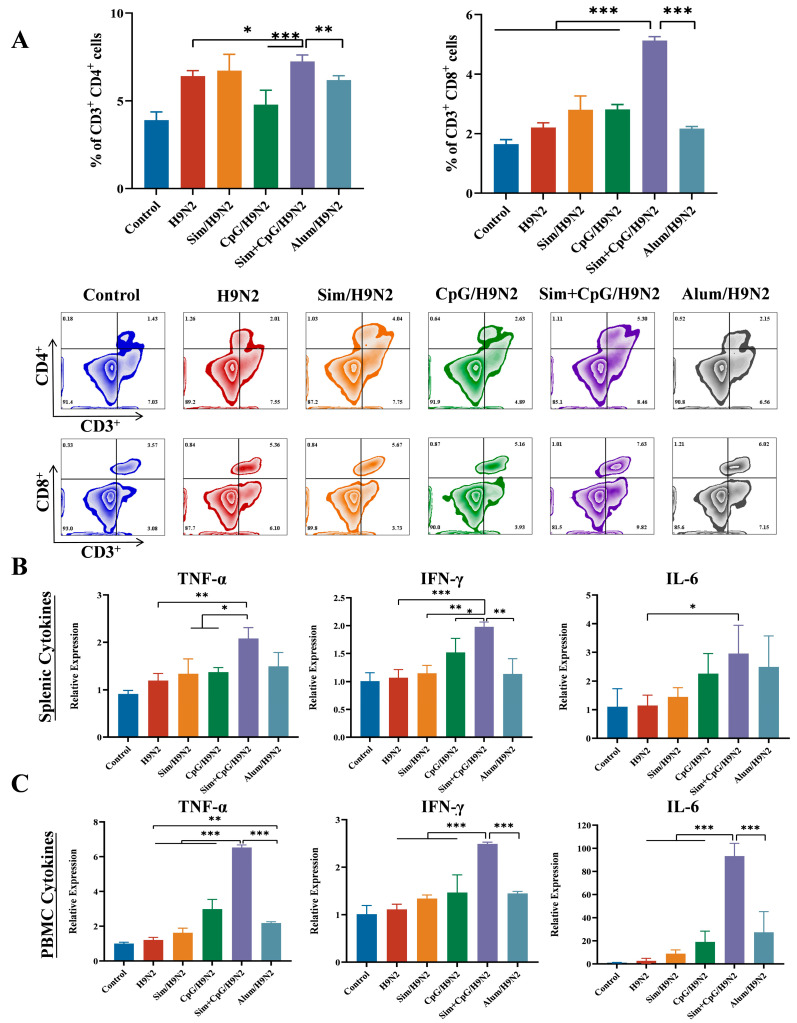
Differentiation of peripheral blood lymphocytes and cytokine secretion after antigen re-stimulation. (**A**) Proportions of CD3^+^CD4^+^ T cells and CD3^+^CD8^+^ T cells, along with flow cytometry scatter plots. All Cytokine levels were measured by quantifying mRNA expression using qRT-PCR. (**B**) Cytokine levels in the spleen at 21 days post-primary immunization. (**C**) Cytokine levels in PBMCs at 21 days post-primary immunization. All data are presented as mean ± standard deviation. Statistical significance is defined as * *p* < 0.05, ** *p* < 0.01, *** *p* < 0.001.

CD4 T cells primarily activate and coordinate other immune cells by releasing signaling molecules, thereby driving adaptive immune responses [20]. CD8 T cells can directly recognize and destroy virus-infected cells, cancer cells, or other abnormal cells [21]. These two types of T cells work together to eliminate pathogens and maintain immune balance. Based on the above results, we further analyzed the lymphocyte subpopulations of PBMCs (Figure 2A). Flow cytometry analysis showed that, 48 h after antigen re-stimulation, the proportions of CD3^+^CD4^+^ T cells and CD3^+^CD8^+^ T cells in the Sim + CpG/H9N2 group were significantly higher than those in the H9N2 and Alum/H9N2 groups (Figure 3A). The increased ratios of these cells indicate that Sim + CpG/H9N2 promotes lymphocyte differentiation and enhances the cellular immune response to the vaccine.

Cytokines are “chemical messengers” secreted by immune cells to coordinate, activate, and regulate immune responses [22]. IFN-γ is mainly secreted by activated Th1 cells and NK cells, driving macrophage activation and antigen presentation, thereby enhancing cellular immune responses [23]. TNF-α and IL-6 are core mediators of the acute-phase response, with TNF-α promoting the amplification of the inflammatory cascade, while IL-6 enhances T cell differentiation [24]. To assess the effects of Sim + CpG/H9N2 on splenic and PBMC immune responses, we analyzed the mRNA expression levels of key cytokines were quantified in splenic tissue and PBMCs on day 21 following primary immunization. Compared to other groups, Sim + CpG/H9N2 induced elevated levels of the inflammatory cytokines IFN-γ, TNF-α, and IL-6 in both the spleen and PBMCs (Figure 3B,C). This suggests that Sim + CpG/H9N2 can systematically activate the inflammatory response of splenic and peripheral immune cells.

### 3.3. Sim + CpG/H9N2 Promotes the Expression of Genes Related to Germinal Centers

**Figure 4:**
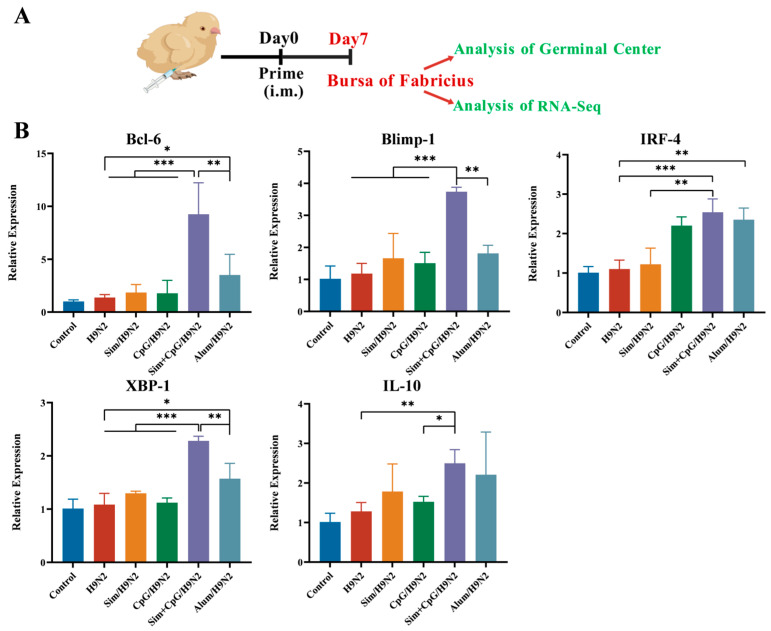
Expression of germinal center-related cytokines. (**A**) Immunization schedule and sampling time. (**B**) On day 7 post-immunization, bursa from each group was collected to evaluate the mRNA expression levels of AID, BCL6, Blimp-1, IRF4, and XBP1. All data are presented as mean ± standard deviation. Statistical significance is defined as * *p* < 0.05, ** *p* < 0.01, *** *p* < 0.001.

After vaccination, the formation of germinal centers (GCs) is important for the development of persistent humoral immunity to the vaccine. GCs protect the body by producing long-lived B cells and plasma cells that generate high-affinity antibodies [25]. Given the high expression of antibodies, we evaluated the impact of the vaccine on GC formation (Figure 4A). In the GC response, Bcl-6 acts as a core transcription factor. It drives the formation and maintenance of GC B cells and promotes their proliferation [26]. The H9N2 inactivated vaccine did not upregulate Bcl-6, indicating limited regulation of GC formation. In contrast, Sim + CpG/H9N2 significantly induced Bcl-6 upregulation, suggesting a positive effect on GC formation (Figure 4B).

In the later stages of the GC response, Blimp-1, IRF-4, and XBP-1 induce B cell differentiation into plasma cells. These plasma cells produce antibodies and migrate to the bone marrow for sustained production. Memory B cells, on the other hand, retain antigen specificity for rapid recall [27]. IL-10 helps maintain the GC microenvironment by preventing excessive inflammation and promoting B cell survival, thus preventing autoimmune reactions [28]. The Sim + CpG/H9N2 vaccine increased the mRNA expression of Blimp-1, IRF-4, XBP-1, and IL-10, indicating its ability to drive B cell differentiation (Figure 4B). These results suggest that Sim + CpG is more effective than traditional Alum in inducing GC formation. This lays the foundation for producing long-lasting, high-affinity antibodies and improving vaccine immunogenicity.

### 3.4. The Immune Response Mechanism of Sim + CpG/H9N2

To investigate the mechanism by which the combination of Simvastatin (Sim) and CpG as adjuvants enhances the immune response of the H9N2 inactivated vaccine, we collected Bursa of Fabricius tissue 7 days post-primary vaccination for RNA-sequencing analysis (Figure 4A).

**Figure 5:**
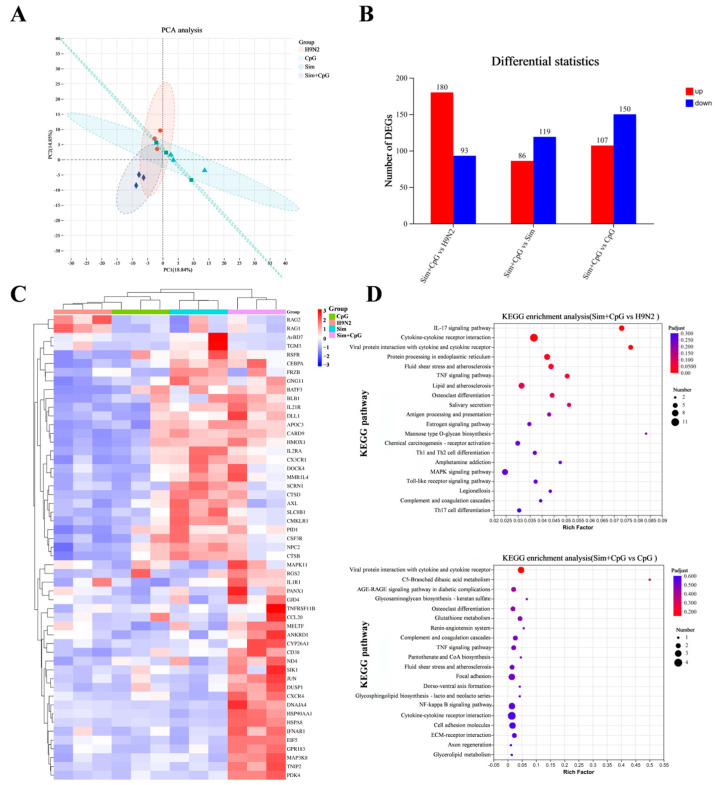
RNA-sequencing analysis of bursa of fabricius tissue 7 days post-immunization. (**A**) PCA analysis between samples. (**B**) Histogram of differential gene analysis of transcripts. (**C**) Clustering analysis of differentially expressed genes in the transcriptome. (**D**) KEGG enrichment analysis (Sim + CpG/H9N2 vs. H9N2, Sim + CpG/H9N2 vs. CpG/H9N2).

Compared to H9N2 alone, the Sim + CpG/H9N2 group exhibited 180 upregulated genes and 93 downregulated genes. Compared to the CpG/H9N2 group, the Sim + CpG/H9N2 group showed 107 upregulated genes and 150 downregulated genes. Compared to the Sim/H9N2 group, the Sim + CpG/H9N2 group showed 86 upregulated genes and 119 downregulated genes (Figure 5A,B). Gene clustering analysis indicated that, compared to other group, Sim + CpG/H9N2 significantly upregulated genes such as CXCR4, CCL20, IL21R, CD38, TNF, and IFN. This suggests that the combined use of Sim and CpG plays a crucial role in antigen presentation, germinal center formation, and regulation of inflammatory factors, thereby enhancing the vaccine’s immune efficacy (Figure 5C).

KEGG pathway enrichment analysis revealed that, compared to H9N2 and CpG/H9N2, Sim + CpG/H9N2 was enriched in pathways such as TNF signaling, cytokine-receptor interaction, NF-κB signaling, and cell adhesion molecules (CAMs), significantly enhancing antiviral capacity and improving vaccine immunogenicity (Figure 5D).

**Figure 6:**
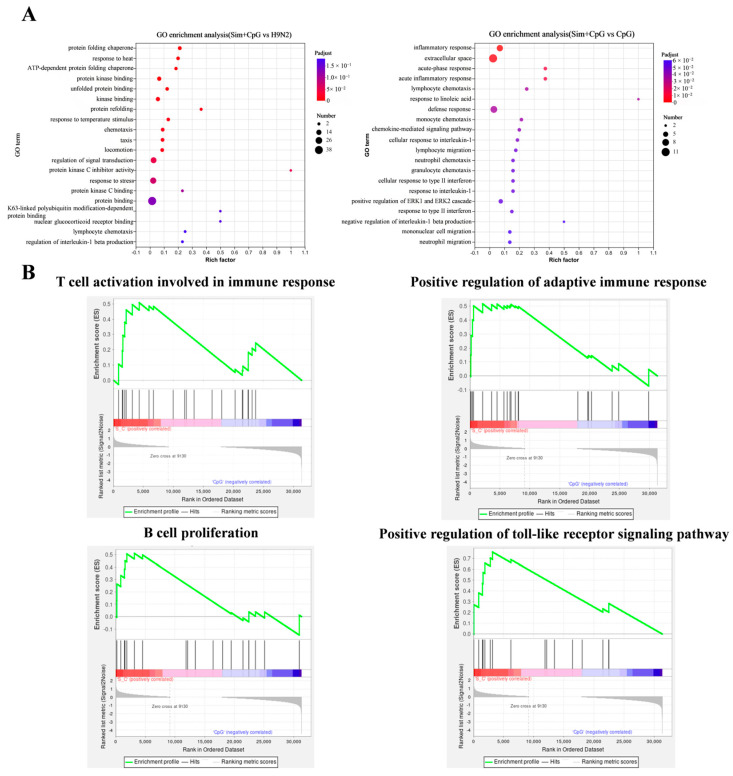
RNA-sequencing analysis of bursa of fabricius tissue 7 days post-immunization. (**A**) GO enrichment analysis (Sim + CpG/H9N2 vs. H9N2, Sim + CpG/H9N2 vs. CpG/H9N2). (**B**) GESA analysis (Sim + CpG/H9N2 vs. CpG/H9N2).

Additionally, GO enrichment analysis results show that Sim + CpG/H9N2 is enriched in pathways related to stress response activation, immune cell recruitment, and inflammatory signaling. This is achieved through the activation of the heat shock protein pathway, which enhances antigen presentation. It also promotes chemokine-mediated immune cell recruitment, such as neutrophil migration, and amplifies the IL-1/ERK inflammatory signaling cascade. These processes create a “stress-recruitment-response” synergistic network, significantly enhancing the immunogenicity of the H9N2 vaccine (Figure 6A). Further gene set enrichment analysis (GSEA) revealed that, compared to CpG/H9N2, Sim + CpG/H9N2 promoted T and B cell activation and proliferation. This enhancement improves adaptive immune responses (Figure 6B).

Overall, these findings show that Sim + CpG, through its synergistic effects, not only boosts innate immunity (by activating inflammatory responses, complement systems, and TLR signaling) but also activates adaptive immunity (through enhanced antigen presentation and T cell differentiation). This significantly increases the antiviral capacity and protective efficacy of the H9N2 inactivated vaccine, offering valuable insights for vaccine design.

### 3.5. Development of Immune Organs and Biosafety After Vaccination

**Figure 7:**
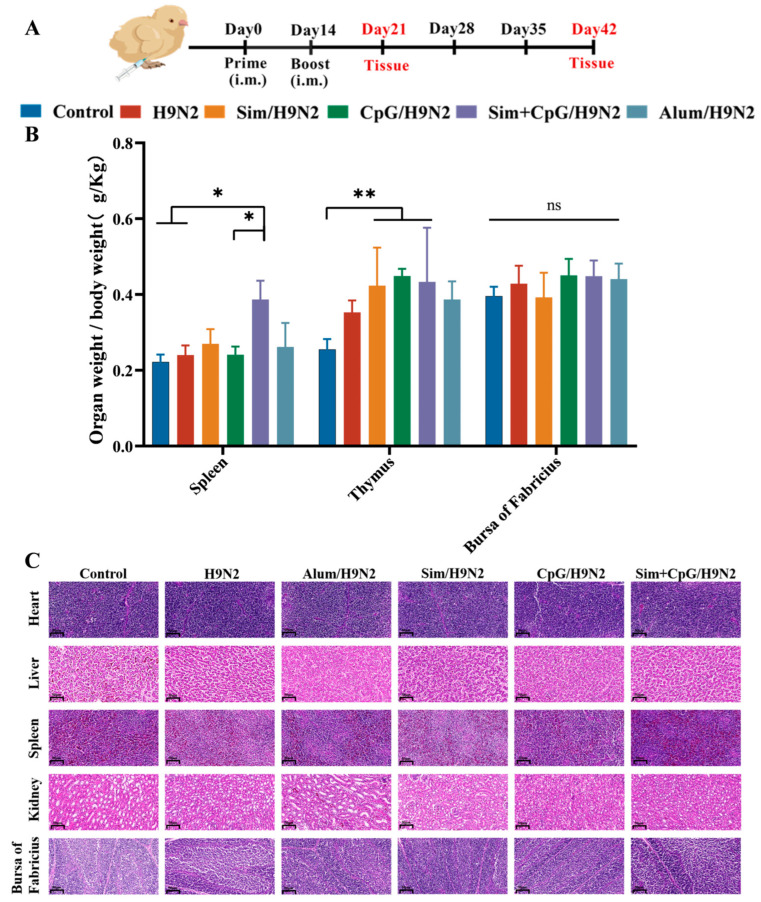
Development of immune organs and biosafety after vaccination. (**A**) Vaccination schedule and tissue collection time points. (**B**) Proliferation indices of the spleen, thymus, and bursa of Fabricius. (**C**) Histopathological examination of the heart, liver, spleen, kidneys, and bursa of Fabricius collected on day 42 post-immunization using H&E staining(scale bar = 50 μm). All data are presented as mean ± standard deviation. Statistical significance is defined as ns (no significance), * *p* < 0.05, ** *p* < 0.01.

The development of immune organs provides the structural foundation for establishing and regulating immune responses [29]. Immune organ indices offer valuable insights into immune activation. To assess the impact of Sim + CpG on immune function, we collected central immune organs (thymus, bursa of Fabricius) and peripheral immune organs (spleen) [30,31,32] from the animals 21 days after primary immunization (Figure 7A). We weighed the organs and calculated the proliferation index. Compared to the H9N2 group, the spleen index was significantly higher in the Sim + CpG/H9N2 group. The thymus and bursa of Fabricius indices showed an upward trend (Figure 7B). These findings suggest that Sim + CpG promotes the development of T cells and B cells, enhancing the vaccine’s immunogenicity.

Vaccine safety is a key consideration in vaccine design and adjuvant selection. To evaluate the safety of Sim + CpG/H9N2, histopathological analysis was conducted on spleen, thymus, bursa of Fabricius, liver, and kidney tissues collected at 42 days post-primary immunization (Figure 7C). H&E staining showed that the organ structures in the Sim + CpG/H9N2 group were intact, with no pathological abnormalities. This indicates that Sim + CpG is safe as an adjuvant for the H9N2 vaccine.

## 4. Discussion

We evaluated the effects of Sim and CpG as adjuvants on the H9N2 vaccine using multiple immunological indicators. The results showed that the combined use of Sim and CpG significantly enhanced both humoral and cellular immune responses, promoted the development of immune organs, increased the expression of germinal center-associated genes, improved the immunogenicity of the H9N2 inactivated vaccine.

First, antibodies are a crucial indicator for evaluating vaccine efficacy [16,17]. The addition of Sim and CpG as adjuvants to the H9N2 vaccine significantly increased IgY and HI antibody levels, indicating that Sim + CpG/H9N2 provides strong antibody-mediated protection.

Cellular immune responses are essential for clearing intracellular infections, providing long-term memory, countering pathogen mutations, and supporting cancer immunity [18]. Sim + CpG/H9N2 significantly stimulates lymphocyte proliferation, providing a foundation for comprehensive, durable, and mutation-resistant vaccine protection [20]. Additionally, increased CD4+/CD8+ T-cell ratios and upregulation of cytokines such as IFN-γ, TNF-α, and IL-6 indicate that Sim + CpG/H9N2 plays a key role in pathogen clearance and maintaining immune homeostasis [21,22,23,24].

The formation of germinal centers (GCs) is critical for inducing long-lasting humoral immunity [25]. Given the elevated IgG and HI antibody levels described above, we evaluated the vaccine’s effect on GC formation. Upregulation of AID, BCL6, Blimp-1, IRF4, and XBP1 indicates that the vaccine promotes GC formation and activation, providing a basis for generating persistent, high-affinity antibodies and enhancing vaccine immunogenicity [27].

RNA-sequencing analysis of the bursa of Fabricius further revealed the molecular mechanisms underlying vaccine-induced immune activation. Differential gene expression, GO, and KEGG pathway enrichment analyses showed that the Sim + CpG adjuvant synergistically enhances both innate immunity (inflammation, complement system, TLR signaling) and adaptive immunity (antigen presentation, T-cell differentiation) through multiple pathways. This substantially improves the antiviral efficacy and protective capacity of the H9N2 inactivated vaccine.

In addition, enlargement of immune organs is generally associated with immune cell proliferation and activation [29]. Increased organ indices of the spleen, thymus, and bursa of Fabricius further confirm the immune-stimulating effects of the vaccine.

In summary, Sim + CpG/H9N2 induces a comprehensive immune response. It promotes antibody production, T-cell activation, and germinal center formation. It also regulates molecular responses in immune organs. These effects significantly enhance the antiviral efficacy and protective capacity of the H9N2 inactivated vaccine.

Challenge experiments with the virus can provide direct evidence of protection and further strengthen the study conclusions. Although our results demonstrate that Sim + CpG/H9N2 enhances the antiviral efficacy and protective capacity of the H9N2 inactivated vaccine, we acknowledge that the absence of challenge experiments is a limitation of this study. Due to biosafety level restrictions and the scope of the current work, such experiments could not be performed. The immunological indicators evaluated in this study, including HI antibody titers and cellular immune responses, are widely recognized as reliable measures of protection in avian influenza vaccine research. In future studies, we plan to conduct H9N2 virus challenge experiments to further validate the protective effects of the Sim + CpG/H9N2 vaccine.

## 5. Conclusions

In summary, Sim + CpG/H9N2 significantly increased the H9N2-specific IgY antibody titers and HI antibody titers. It also promoted the formation of germinal centers, lymphocyte proliferation, differentiation, and the development of immune organs. These effects effectively enhanced both humoral and cellular immune responses to the vaccine. Additionally, RNA-sequencing analysis of the bursa of Fabricius revealed significant enrichment of immune-related genes. This not only enhanced innate immunity but also activated adaptive immunity. As a result, the antiviral efficacy and protective capacity of the H9N2 inactivated vaccine were significantly improved. These findings provide new insights for the design and use of seasonal Avian vaccines.

## Figures and Tables

**Table 1:** The primer sequences.

Gene	Sense Strand (5′−3′)	Antisense Strand (5′−3′)
IL-4	GTGCCCACGCTGTGCTTAC	AGGAAACCTCTCCCTGGATGT
IL-6	AAATCCCTCCTCGCCAATCT	CCCTCACGGTCTTCTCCATAAA
IFN-γ	ACGACACCATCCTGGACACC	TTTGGCGTTGGCTGTCGTTC
IL-1β	CATCACCAACCAACCCGA	ACGAGATGGAAACCAGCAA
TNF-α	CAGCCCTCACATCACCTC	GCTGCCACTCCAGCAATA
IL-10	CGCTGTCACCGCTTCTTCA	TCCCGTTCTCATCCATCTTCTC
CD40	GGGCTCGTGGTGAAGGTGAAAG	GGATCAGCACTGACAGCGATGAG
BCL6	GCAGTTCAGAGCCCACAAAA	GTTCAGACGGGAGGTGTACA
Blimp-1	ACACAGCGGAGAGAGACCAT	GCACAGCTTGCACTGGTAAG
IRF4	GTGTGGGAGAATGACGAGAAG	AAGGAGATGTGATTGGGAAGG
XBP1	GTGCGAGTCTACGGATGTGA	GTGCGAGTCTACGGATGTGA
β-actin	GAGAATTGTGCGTGACATCA	CCTGAACCTCTCATTGCCA

## Data Availability

The original contributions presented in this study are included in the article. Further inquiries can be directed to the corresponding author.
